# Audiological Features in Patients with Rheumatoid Arthritis: A Systematic Review

**DOI:** 10.3390/ijms252413290

**Published:** 2024-12-11

**Authors:** Jiann-Jy Chen, Chih-Wei Hsu, Yen-Wen Chen, Tien-Yu Chen, Bing-Syuan Zeng, Ping-Tao Tseng

**Affiliations:** 1 Prospect Clinic for Otorhinolaryngology & Neurology, Kaohsiung 811026, Taiwan; jiannjy@yahoo.com.tw (J.-J.C.); kevinachen0527@gmail.com (Y.-W.C.); 2 Department of Otorhinolaryngology, E-Da Cancer Hospital, I-Shou University, Kaohsiung 824005, Taiwan; 3 Department of Psychiatry, Kaohsiung Chang Gung Memorial Hospital and Chang Gung University College of Medicine, Kaohsiung 833401, Taiwan; harwicacademia@gmail.com; 4 Department of Psychiatry, Tri-Service General Hospital, School of Medicine, National Defense Medical Center, Taipei 114202, Taiwan; verducciwol@gmail.com; 5 Institute of Brain Science, National Yang Ming Chiao Tung University, Taipei 112, Taiwan; 6 Institute of Biomedical Sciences, National Sun Yat-sen University, Kaohsiung 804201, Taiwan; 7 Department of Internal Medicine, E-Da Cancer Hospital, I-Shou University, Kaohsiung 824005, Taiwan; 8 Department of Psychology, College of Medical and Health Science, Asia University, Taichung413305, Taiwan; 9 Institute of Precision Medicine, National Sun Yat-sen University, Kaohsiung 804201, Taiwan

**Keywords:** rheumatoid arthritis, cochleopathy, hearing impairment, neuropathy, ossicles, steroid

## Abstract

Hearing impairment in patients with rheumatoid arthritis has been underestimated for decades. Rheumatoid arthritis can affect both the middle ear (specifically, the incudomalleolar and incudostapedial joints) and inner ear (including the cochlea and acoustic nerve) simultaneously. Despite ongoing research, consensus on effective treatments for hearing impairment in these patients remains elusive. This systematic review aims to consolidate clinically relevant information for healthcare providers by summarizing current evidence on hearing impairment in rheumatoid arthritis patients. We conducted the current systematic review by searching platforms of PubMed, Embase, ClinicalKey, Web of Science, and ScienceDirect to retrieve eligible articles regarding hearing impairment related to rheumatoid arthritis. We extract any data on characteristics, pathophysiology, examination, and treatment related to rheumatoid arthritis. Based on the currently available evidence, we advocate for the use of specific audiometric tests to facilitate early detection of hearing impairment in these patients. Regular audiological assessments are recommended to monitor hearing ability and potentially prevent further deterioration. Finally, we propose a modified treatment protocol that integrates steroids, hydroxychloroquine, and non-invasive brain stimulation as a novel therapeutic approach for managing these symptoms. This protocol aims to offer clinicians new strategies to address hearing impairment in patients with rheumatoid arthritis effectively.

## 1. Introduction

Rheumatoid arthritis (RA) ranks among the most prevalent autoimmune diseases, affecting approximately 0.46% of the population (95% confidence interval: 0.39% to 0.54%) [1]. It is characterized by painful inflammation of single or multiple joints, leading to erosive bone damage [2]. This inflammatory response is believed to involve the recruitment of leukocytes, particularly CD4+ T cells and monocytes, into affected tissues [3]. Activated CD4+ T cells stimulate macrophages and fibroblasts, promoting pannus formation and subsequent erosion of bone and cartilage structures [4], ultimately resulting in chronic joint degradation [5]. Theoretically, joints in the ear, nose, and throat regions could be potential targets of RA. Specifically, the incudomalleolar and incudostapedial joints, being true diarthroses, are implicated as potential sites affected in patients with rheumatoid factors [6,7,8], previously termed as oto-arthritis [9,10,11]. This hypothesis finds support in clinical observations. For instance, compared to non-RA subjects with sensorineural hearing loss, those with RA generally exhibit a poorer prognosis [12]. Additionally, among individuals with various autoimmune conditions, only those with RA show a significantly higher incidence of sensorineural hearing loss compared to controls [13]. Conversely, a study examining otological symptoms (otalgia, hearing loss, tinnitus, and vertigo) in RA patients reported significantly higher incidences in the patient group compared to controls (*p* = 0.001) [14]. Furthermore, a recent meta-analysis highlighted a prevalence of 16.14% (95% confidence interval: −9.03% to 41.31%) for sensorineural hearing loss in RA cases [15]. Specifically, Lee et al. demonstrated an age- and gender-specific relationship between RA and sensorineural hearing loss, with significantly higher hazard ratios observed among individuals aged 50 years or older and in the male subgroup [16]. These age- and gender-specific risk factors are corroborated by other large-scale studies [17,18], although the male predominance was not consistently observed in all studies [19]. Conversely, cumulative disease activity has been identified as a predictive factor for hearing impairment in RA patients [20]. Conversely, several reports have indicated a significantly higher prevalence of RA or positive rheumatoid factors in patients with sensorineural hearing loss [21,22,23]. In a recent review article addressing the systemic nature of rheumatoid arthritis in hearing function [24], they noticed a heterogeneity in the nature (type of hearing loss), degree, as well as configuration of the hearing loss. Among the affected patients, the sensorineural hearing loss mainly occurred in ultrahigh or high frequencies [24], which might be related to the systematically increased ototoxicity as a leading cause of hearing loss in this population.

However, the involvement of the incudomalleolar and incudostapedial joints in patients with RA was not as prevalent as previously anticipated [25,26,27,28,29,30]. Instead, sensorineural hearing loss related to cochlear and neuropathic mechanisms appears to be more common [20,31,32,33]. While inner-ear involvement is frequently reported in other autoimmune diseases such as systemic lupus erythematosus [34,35,36], systemic sclerosis [37,38], and anti-phospholipid syndrome [39], relatively few studies have addressed inner-ear diseases specifically related to RA [40,41,42,43,44,45,46]. Similar to other autoimmune ear disorders, the exact cause and pathophysiology remain elusive, with hypotheses including excessive autoantibody production, direct autoimmune attacks, microcirculatory disturbances, destruction of ossicular joints [4,47], and ototoxicity from medications used in RA treatment, such as methotrexate [48] and leflunomide [49].

Given the unclear pathophysiology, no definitive examination has been established to detect hearing impairment associated with RA [24,50]. Current tools, including audiograms, otoacoustic emissions, C-reactive protein levels, erythrocyte sedimentation rates, and rheumatoid factors, lack specificity and sensitivity [6,51]. While researchers have suggested potential roles for plasma matrix metalloproteinases (MMPs) [52] and cochlin [47] in detecting hearing impairment linked to RA, conclusive results have not yet been achieved.

Insufficient recognition results in ineffective treatment. Currently, there is no consensus on the efficacy of treatments for hearing impairment in patients with RA. Given its autoimmune nature, various immune-modulating agents such as hydroxychloroquine, methotrexate, cyclosporine, azathioprine, infliximab, etanercept, and corticosteroids are prescribed for these patients. However, concerns about potential toxicity from these agents [53,54,55,56,57] have deterred clinicians from prescribing them at adequate dosages. Not only is there apprehension about ototoxicity and other adverse effects, but also insufficient knowledge about determining the “appropriate dosage” limits the use of immune-modulating agents in such cases [51].

The goal of this systematic review is to summarize the existing evidence on hearing impairment in patients with RA. To improve the application of treatments for managing auditory dysfunction in these patients, we present a modified treatment protocol that includes steroids, hydroxychloroquine, and non-invasive brain stimulation.

## 2. Methods and Materials

**Figure 1:**
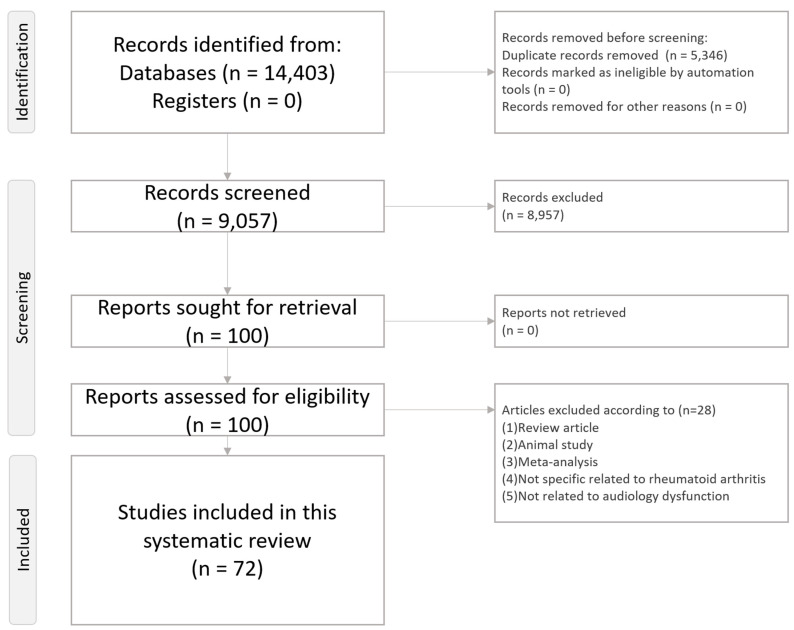
PRISMA2020 Flowchart of current systematic review. Figure 1 illustrates the flowchart outlining the procedure of the present systematic review.

This systematic review adheres to the Preferred Reporting Items for Systematic Reviews and Meta-Analyses (PRISMA) statement (Table S1 and Figure 1) [58]. The review protocol was registered on the INPLASY platform (INPLASY202460125, https://inplasy.com/inplasy-2024-6-0125/), accessed on 21 January 2024.

### 2.1. Literature Search Strategy

Electronic searches were conducted on PubMed, Embase, ClinicalKey, Web of Science, and ScienceDirect. Detailed search strategies and keywords for each platform are provided in Table S2. Additionally, a manual search of reference lists from the included articles was performed. The initial search was conducted on 19 January 2024, with the final update search completed on 21 June 2024. In cases where data were insufficient in the original papers, the corresponding authors were contacted via email to request additional information.

### 2.2. Inclusion and Exclusion Criteria

This systematic review focused on audiological issues related to RA, encompassing characteristics, pathophysiology, examination, and treatment. Inclusion criteria were as follows: (a) studies investigating audiological issues in RA; (b) case reports/series, observational trials, case-control trials, or randomized controlled trials; and (c) studies involving patients diagnosed with RA.

Exclusion criteria were: (a) studies that did not include patients with RA; (b) studies that did not address characteristics, pathophysiology, examination, or treatment related to audiological dysfunction in RA; and (c) animal studies. Review articles were utilized for manual extraction of relevant articles from their reference lists, with excluded studies documented in Table S3.

### 2.3. Article Screening Process

Following electronic searches across all five databases and the initial application of inclusion/exclusion criteria based on title and abstract, eligible articles underwent full-text examination. Duplicate articles were manually removed, and the remaining articles underwent full-text screening to determine final inclusion.

### 2.4. Data Extraction

Data extraction was conducted by PT Tseng, who performed full-text examinations to extract information on characteristics, pathophysiology, examination, and treatment related to RA.

### 2.5. Article-Quality Grading

The quality of clinical studies was evaluated using the Newcastle–Ottawa Scale by JJ Chen and PT Tseng (Table S4) [59]. In brief, the average score of the included studies was 4.1 (maximum: 8) according to the Newcastle–Ottawa Scale. The average score of each item was: 1.0 (Case definition), 0.8 (Representativeness), 0.5 (Control selection), 0.5 (Control definition), 0.4 (Comparability), 0.8 (Ascertainment), 0.03 (Same method), and 0.03 (Non-Response rate).

## 3. Characteristics and Features of Audiology Presentation in RA

**Figure 2:**
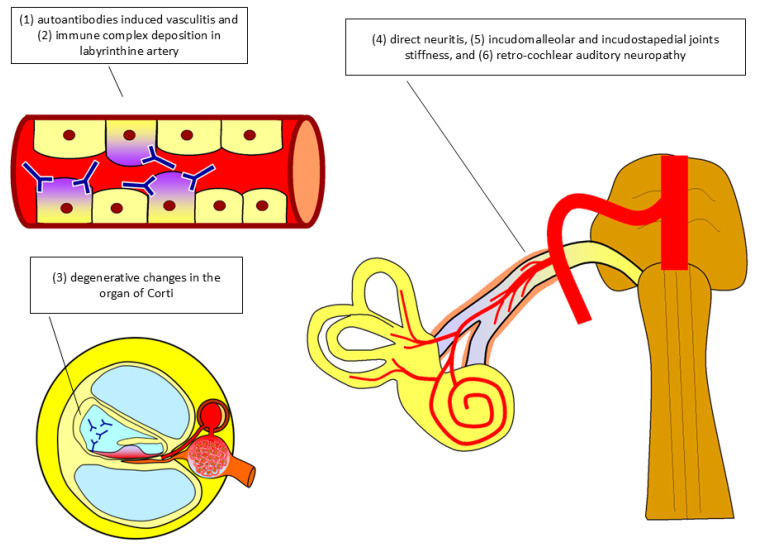
Schematic diagram of the physiopathology of rheumatoid arthritis in audiology dysfunction. Figure 2, which was drawn by the first author, illustrates the pathophysiology of rheumatoid arthritis-related antibodies and the formation of immune reactions contributing to audiological dysfunction. It overall consisted of six mechanisms, including (1) autoantibodies-induced vasculitis, (2) immune complex deposition in the labyrinthine artery, (3) degenerative changes in the organ of Corti, (4) direct neuritis, (5) incudomalleolar and incudostapedial joints stiffness, and (6) retro-cochlear auditory neuropathy.

The overall pathophysiological response involving audiological dysfunction related to RA is illustrated in Table 1 and Figure 2.

### 3.1. Middle-Ear Involvement Subtype

#### 3.1.1. Characteristics of Middle-Ear Involvement Subtype

The prevalence of conductive hearing loss in patients with RA ranges from 1.9% to 24.3% [19,25,26], which is relatively low compared to sensorineural hearing loss [60,61]. The incidence of right-side and left-side conductive hearing loss is similar (5% vs. 2%, respectively) [62]. Additionally, the severity of hearing impairment correlates positively with the Steinbrocker functional index, an indicator of RA severity [26].

#### 3.1.2. Physiopathology of Middle-Ear Involvement Subtype

In patients with RA, antigen-activated CD4+ T cells stimulate macrophages and fibroblasts to secrete factors that promote synovial cell proliferation, leading to chronic inflammation and contributing to joint destruction [5]. TNF, a dominant proinflammatory cytokine in RA [63], also plays a crucial role in osteoclast differentiation and activation [64]. The incudomalleolar and incudostapedial joints in the middle ear are true diarthroses joints that may be susceptible targets [6,7]. Prolonged involvement of these joints by rheumatoid factors can lead to otosclerosis and result in conductive hearing loss [25]. In addition to stiffness of the ossicular system [65] or inflammatory damage to the tympanic membranes [66], decreased stability of ligamentous anchorage also contributes significantly to conductive hearing impairment in RA patients [33]. Furthermore, the dissolution of disk material and the formation of pannus-like tissue during proliferation of synovial-type elements have been reported in the ossicular joints of RA patients [67]. Consequently, bilateral high-frequency hearing impairment is observed in these patients due to these pathological changes in the ossicular joints [67]. Rheumatoid nodules, common extra-articular features of RA, can also contribute to hearing impairment in these patients [68]. These nodules most frequently occur at subcutaneous pressure points but can also affect sites in the ear, nose, and throat, potentially obstructing the auditory conducting process [68].

Another characteristic of RA is complicated vasculitis, which can lead to decreased blood perfusion of the ossicles, particularly affecting the long process of the incus and potentially resulting in ossicle necrosis [6].

Another issue related to conductive hearing impairment in patients with RA involves increased stiffness of the ossicular system [5,26]. However, this damage may not always be significant enough to be detected by routine pure tone audiometry [52]. Since the incudomalleolar and incudostapedial joints are crucial for sound transmission and can become functionally fixed, stiffness in these joints might not necessarily disrupt sound conduction to the cochlea [4,5]. As a result, patients with RA may not subjectively notice hearing problems in their daily lives, despite potentially having impaired hearing. However, the damage is progressive, underscoring the importance of regular check-ups for hearing ability in these patients to detect and prevent future deterioration.

#### 3.1.3. Examination for Middle-Ear Involvement Subtype

In patients with RA, a significantly elevated ipsilateral stapes reflex threshold value (up to 1.0 kHz) may indicate early signs of conductive hearing loss due to ossicular joint stiffness and fibrosis [4]. In advanced cases, multiple-frequency tympanometry [6], which involves sweeping the probe tone across a range of frequencies (e.g., from 250 to 2000 Hz), can assess the resonant frequency of the middle ear system and diagnose pathology in the adult ossicular chain [4]. Additionally, higher-frequency probe-tone tympanometry can more accurately detect middle ear effusion [69]. Approximately 40% of RA patients exhibit abnormal resonance values, either increased or decreased [6]. Normal mean air-conduction thresholds have been observed in patients with RA [6], possibly indicating impaired protective mechanisms of the middle ear against high static pressures in these patients [6]. This abnormal finding could explain the discrepancy between normal hearing ability and increased middle ear stiffness [4].

On another note, plasma MMPs play a crucial role in breaking down extracellular matrix proteins during tissue remodeling, both in normal physiological processes and in disease processes [70]. Therefore, plasma MMPs are important in several autoimmune diseases that target the extracellular matrix, such as RA [52]. Evidence suggests MMPs contribute to inner-ear cell damage through oxidative processes [5,68] and the degradation of incudomalleolar and incudostapedial joints [52]. Elevated levels of matrix metalloproteinase-3 (MMP-3) in rheumatoid synovial fluid may predict joint destruction [71], reflecting MMP overexpression in isolated synovium and cartilage of RA patients [72].

### 3.2. Inner-Ear Involvement Subtype

#### 3.2.1. Characteristics of Inner-Ear Involvement Subtype

Conversely, there is increasing documentation of inner-ear damage among patients with RA [4,47,73,74,75]. In addition to rare complications such as acoustic neuroma [76], the cochlea is predominantly affected in the inner-ear involvement subtype [47,77]. The prevalence of sensorineural hearing loss related to inner-ear involvement has been reported as 24–72% in RA patients [20,31,32]. A recent meta-analysis revealed that these patients have nearly four times higher odds of developing sensorineural hearing loss compared to controls [78]. However, there is no significantly increased risk of conductive or mixed hearing loss in RA patients compared to controls [78]. The prevalence of sensorineural hearing loss slightly varies between the left (55%) and right (61%) ears [62], with a high percentage showing bilateral ear involvement [24,32]. While hearing loss can affect all frequencies [52], RA primarily damages high frequencies, specifically 4000–8000 Hz [79], 8000 Hz [43], or 10,000–16,000 Hz [80]. Increased hearing thresholds are notably observed during the acute phase of RA but not during remission [81]. Ozturk et al. noted that the duration of the disease correlates with impaired hearing function, progressing from higher frequencies (10,000 Hz) in the early stages (1–5 years) to lower frequencies (4000 Hz) in the intermediate stages (6–10 years), and ultimately affecting all frequencies (11–15 years) [82]. Another study reported sensorineural hearing loss percentages of 18%, 19%, and 57% in low, mid, and high frequencies, respectively [62], suggesting a predominant high-frequency pattern possibly due to impaired protective mechanisms in the inner ear following ossicular joint fixation [6]. This high-frequency predominance [31] may serve as an early indicator of RA-related hearing impairment, occurring before middle and low frequencies are affected [73]. Moreover, most RA-related hearing losses are categorized as slight (34%), mild (12%), moderate (9%), severe (1%), and profound (2%) [62]. Additionally, patients with RA tend to experience more severe hearing loss compared to those without the condition; for instance, patients with sudden sensorineural hearing loss and RA exhibit higher levels of hearing loss, greater rates of profound hearing loss, and poorer treatment responses compared to those without RA [83]. Beyond abnormal hearing thresholds, patients with RA also demonstrate significantly impaired speech-discrimination scores compared to controls [84].

#### 3.2.2. Physiopathology of Inner-Ear Involvement Subtype

As mentioned earlier, TNF–alpha plays a pivotal role in RA by enhancing osteoclast differentiation and activation [64]. Recent studies have observed that under specific concentrations, TNF–alpha can penetrate the inner ear [85,86,87], leading to disturbances in cochlear microcirculation [88] by actively constricting capillaries [89].

In addition to vasculitis, there is evidence supporting direct neuritis [32] or degenerative changes in the organ of Corti [40] related to the autoimmune process of RA. Furthermore, immune complex deposition in the labyrinthine artery associated with RA could contribute to arterial stenosis or occlusion in the cochlea, which has limited collateral circulation [73]. Consequently, ischemia in the cochlea may result in profound sensorineural hearing impairment even with slight disruptions in blood supply [73]. This hypothesis finds partial support in studies showing a significant association between atherosclerosis and sensorineural hearing impairment in RA patients [60].

While cochleopathy is recognized as a primary target organ in RA-related hearing impairment, neuropathy may also contribute to sensorineural hearing impairment in these patients. Specifically, retro-cochlear involvement by RA-related immunological complexes in the endothelium of the vasa vasorum could lead to auditory neuropathy due to impaired blood supply to the cochlear nerve [90].

#### 3.2.3. Examination for Inner-Ear Involvement Subtype

Various otologic examinations are currently available to detect inner-ear damage. For instance, distortion-product otoacoustic emission (DPOAE), which involves simultaneous stimulation of the cochlea by two pure-tone frequencies, serves as a noninvasive tool to assess the functional status of cochlear outer hair cells in different inner-ear diseases [51]. In a recent study investigating DPOAE absence in patients with positive rheumatoid factor, researchers observed a statistically significant association between DPOAE absence and positive rheumatoid factor [91].

Similarly, transient evoked otoacoustic emission (TEOAE), which reflects outer hair cell function, is useful for cochlear evaluation [92]. A decrease in TEOAE may indicate initial inner-ear damage caused by RA [4,93,94]. Moreover, risk factors such as “older age”, “male gender”, “longer disease duration”, and “acoustic trauma” may be associated with increased hearing thresholds and reduced TEOAE [4]. Instead of examining each frequency individually, the average hearing loss value (AHLV) can be used as a statistical alternative to individual frequency values. AHLV, which is less affected by RA drug therapy, shows a statistically significant correlation with disease activity scores, specifically the Disease Activity Score 28, in RA patients [47].

Some RA-related data, such as the Ritchie Articular Index, C-reactive protein levels, erythrocyte sedimentation rate, and platelet counts, could serve as alternative indicators of disease severity and may be inversely correlated with tympanometry compliance values in these patients [4]. Although Cochlin has been considered another promising candidate for evaluating inner-ear impairment in autoimmune inner-ear diseases, IgG antibodies to human Cochlin play a lesser role in hearing impairment in patients with RA [47]. As mentioned earlier, plasma MMPs play a crucial role in inner-ear cell damage through oxidative processes [5,68]. Specifically, plasma MMP-3 has been significantly correlated with hearing impairment (*p* < 0.001) [5]. Elevated plasma MMP-3 levels in RA patients with sensorineural hearing impairment are believed to be associated with systemic inflammation and tissue damage [5]. Significantly higher levels of MMP-3 in plasma were observed in RA patients developing sensorineural hearing impairment [52].

In addition to traditional RA-related antibodies, some other comorbid autoantibodies may also be associated with features of sensorineural hearing loss in RA patients. For example, Lobo and colleagues reported an association between positive anti-citrullinated protein antibodies and sensorineural hearing loss in patients with RA [95]. Another study involving RA patients demonstrated a significant correlation between sensorineural hearing loss and serum anti-cardiolipin antibody levels [18].

### 3.3. Mixed Subtype

#### 3.3.1. Characteristics and Physiopathology of Mixed Subtype

Mixed middle-ear and inner-ear involvement in patients with RA can be attributed to two primary mechanisms. Firstly, when rheumatoid ossicular joint fixation occurs (specifically in middle ear involvement), it impairs the protective mechanism in the inner ear, thereby exposing cochlear hair cells to long-term intrinsic and extrinsic trauma (specifically in inner-ear involvement) [6]. Secondly, evidence suggests that differences in mean air-bone gap values [52] indicate a superimposition of conductive and sensorineural hearing impairments, resulting in a mixed subtype. This mixed subtype encompasses characteristics of both middle-ear and inner-ear damage. The prevalence rate of mixed-subtype hearing impairment is approximately 10.4% in patients with RA [26]. Some clinical studies suggest that mixed-subtype hearing impairment may manifest in later stages of the disease [4].

#### 3.3.2. Examination for Mixed Subtype

Various audiometric tests have been utilized to assess damage to both the middle and inner ear, including high-frequency audiometry (8.0–16.0 kHz), tympanometry, and transient evoked otoacoustic emissions, as mentioned earlier [4].

## 4. Treatment of RA-Related Middle/Inner-Ear Diseases

At present, there is no specific treatment targeting the various subtypes of hearing impairment in patients with RA. Proposed management approaches include a range of medications with diverse mechanisms, such as RA medications, vasodilators, antioxidants, steroids, lifestyle modifications, and surgical interventions.

### 4.1. RA Medications

Concerns have been raised about the potential ototoxicity of RA medications, including TNF blockers [57], hydroxychloroquine [53,54,55], chloroquine [96], salicylate [97,98,99], etanercept, azathioprine, or cyclosporine [56]. However, conclusive evidence on this matter remains elusive. For instance, Toktas recently demonstrated that infliximab, a TNF blocker, does not appear to affect inner-ear hearing function in RA patients [51]. Similarly, Tsirves found no definitive correlation between medications such as hydroxychloroquine, methotrexate, cyclosporine, azathioprine, mycophenolate mofetil, leflunomide, tocilizumab, infliximab, certolizumab, adalimumab, etanercept, corticosteroids, and calculated AHLVs [47]. Rosenberg also commented that drugs like steroids or penicillamine did not show sensorineural abnormalities in recruited patients [100]. Moreover, a study on hearing impairment in RA patients did not find significant associations between hearing loss and NSAIDs or steroids [26]. Animal studies have similarly shown that direct exposure to etanercept does not result in ototoxicity [101], while cyclosporine has been considered effective in managing sudden sensorineural hearing loss [102]. Thus, medication-related ototoxicity may not explain hearing impairments in RA patients taking these medications; these drugs remain potential options for managing RA-related hearing impairment. Conversely, adalimumab, an anti-TNF-α medication, has shown efficacy in managing steroid-resistant sensorineural hearing loss in RA patients [103]. Although not specific to RA, Rahman et al. demonstrated that etanercept improves hearing function and tinnitus in patients with immune-mediated cochleovestibular disorders [104]. Several studies have highlighted the benefits of RA medications like infliximab in treating hearing impairment in autoimmune hearing loss patients [86,87,105,106], particularly when administered at adequate dosages [107]. Methotrexate, starting at 7.5 mg/week and increasing to 25 mg/week over 8 weeks, has also shown some efficacy in improving hearing impairment in RA patients [108].

### 4.2. Steroid

Steroid therapy is well-established in the treatment of various autoimmune diseases [109]. Researchers have reported favorable outcomes in restoring hearing ability with corticosteroid treatment for sensorineural hearing loss in RA patients [66,85,110]. However, these favorable results were not consistently observed in other studies [111,112]. Li et al. recommended that combined intratympanic and oral steroid therapy may be superior to oral steroids alone in terms of hearing gains and recovery rates in RA patients with sudden sensorineural hearing loss [83].

Although there is no definitive consensus on the optimal oral steroid dosage for managing RA-related hearing impairment, Alexander proposed a treatment protocol for autoimmune inner-ear diseases [113]. This protocol involves a two-phase trial: Phase 1 includes a 1-month course of high-dose prednisone (60 mg/day) plus ranitidine for gastrointestinal prophylaxis [113]. Patients who respond to the prednisone challenge proceed to Phase 2, where oral prednisone is gradually tapered over 18 weeks, resulting in a total treatment duration of 22 weeks with an average daily dosage of 30 mg [113]. The response criteria include at least a 15-dB improvement in the pure-tone air-conduction threshold at any frequency in one ear, or a 10-dB improvement at two consecutive frequencies, along with a significant increase in the word identification score [113]. Based on favorable outcomes and acceptable adverse events, the authors concluded that high-dose corticosteroids could be safe and effective for managing autoimmune inner-ear diseases when patients are carefully selected, educated, and monitored [113].

In addition to oral steroid therapy, intratympanic injections of methylprednisolone (0.5 mL of 40 mg/mL, weekly) have been explored as an alternative treatment for RA-related hearing impairment, showing improved hearing in 68.6% of patients in the high-frequency range (over 2000 Hz) [108].

### 4.3. Vasodilator

Alongside RA medications, vasodilator therapy is recommended for patients to mitigate vasoconstriction-related microcirculation disturbances in the inner ear system [114,115]. Wang et al. reported that a treatment protocol combining alprostadil (10 μg/day, prostaglandin E1) with intravenous dexamethasone (10 mg/day) or oral prednisone (1 mg/kg/day) for 10 days improved hearing function in RA patients with sudden sensorineural hearing loss, but not in those with non-sudden sensorineural hearing loss [116].

### 4.4. Antioxidants and Other Regimens with Anti-Inflammatory or Immunomodulating Effects

Narayan et al. demonstrated the beneficial effects of cyclophosphamide and azathioprine in restoring hearing function in the management of sudden hearing loss in a patient with undiagnosed RA [117]. Several antioxidants, including sodium salicylate, vitamin E, and N-acetyl cysteine, can also be considered as adjunctive treatments to protect the inner ears of patients with RA [118]. Furthermore, Chinese herbal medicine with immunomodulatory effects has shown promise in reducing the risk of hearing loss in these patients [119]. Finally, although direct causative studies are lacking, Huang et al. found an association between the prescription of non-steroidal anti-inflammatory drugs and a lower incidence of sensorineural hearing loss in patients with RA [17].

### 4.5. Lifestyle Modification

In addition to pharmacotherapy, lifestyle modifications are crucial for managing RA in patients. For instance, smoking is theorized to have adverse effects on hearing in these patients. Nicotine from smoking causes vasoconstriction, while carbon monoxide reduces oxygen concentration, potentially leading to cochlear ischemia and decreased function [120]. Similarly, alcohol consumption has been linked to harm in cochlear function, particularly affecting outer hair cells and potentially causing sensorineural hearing impairment [121,122]. Therefore, we recommend that patients with RA discontinue smoking and alcohol consumption. Additionally, as highlighted in most treatment guidelines for sensorineural hearing impairment, exposure to noise can cause extensive cochlear damage and subsequent hearing loss [123,124]. Noise-induced cochlear damage is also a concern for patients with RA and can exacerbate existing hearing impairment.

### 4.6. Surgical Intervention

Surgical intervention should be considered for patients with severe conductive hearing impairment, particularly those with middle-ear involvement, or in cases of mixed-subtype hearing impairment. Surgery can aid in restoring the sound-conduction mechanism of the middle ear [26,125].

### 4.7. Modified Multi-Aspect Treatment Protocol for Managing Audiology Dysfunction Related to RA

**Figure 3:**
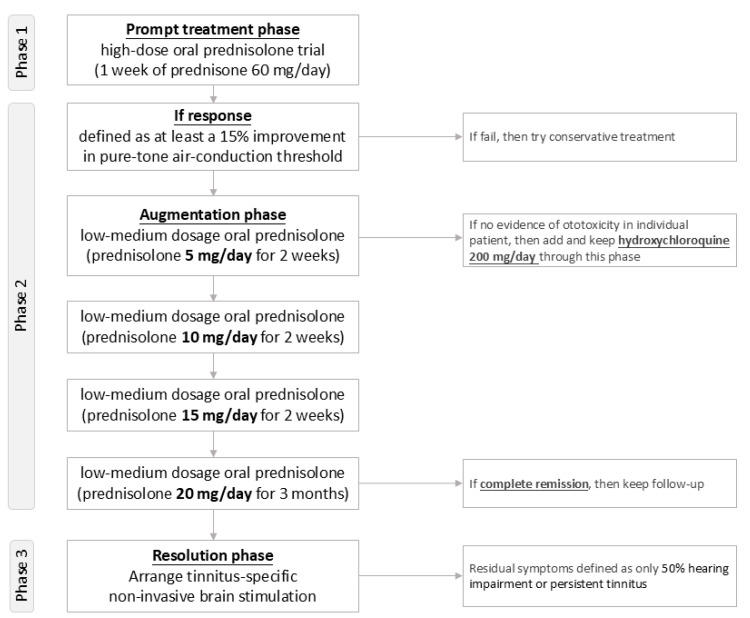
Flowchart of the multi-aspect treatment protocol for managing audiology dysfunction related to rheumatoid arthritis. Figure 3 presents a modified multi-aspect treatment protocol focusing on a 3-phase trial involving steroids, hydroxychloroquine, and non-invasive brain stimulation for managing audiological dysfunction related to rheumatoid arthritis. Note: This is a proposal of a future study protocol.

Based on our preliminary data [126,127], we have developed a modified treatment protocol inspired by Alexander’s approach [113], which consists of three phases for managing audiology dysfunction related to RA (Figure 3).

Phase 1 (Prompt Treatment Phase) involves initiating a high-dose oral prednisolone trial (1 week of prednisone 60 mg/day) to assess the response to steroid treatment. A positive response is defined as at least a 15% improvement in the pure-tone air-conduction threshold.

Phase 2 (Augmentation Phase) targets patients who respond to high-dose steroid therapy. They begin a tapering regimen of oral prednisolone, starting at 5 mg/day for 2 weeks and increasing by 5 mg/day every 2 weeks until reaching a maintenance dosage of 20 mg/day. Hydroxychloroquine 200 mg/day is added throughout the augmentation phase, provided there is no evidence of ototoxicity in the patient.

Phase 3 (Resolution Phase) addresses patients with residual symptoms such as 50% hearing impairment or persistent tinnitus, who undergo non-invasive brain stimulation specifically targeting tinnitus management [128].

However, since this is a proposal of a future study protocol, the proposed treatment protocol requires further validation to assess its effectiveness and applicability in future clinical practice.

### 4.8. Recommendations About Referral to ENT Doctors in Clinical Practice

Although there had not been consensus regarding the red flags or timing for referral to an ENT doctor, we summarized some potential time points for referral consideration. First, patients started to complain of audiological symptoms, such as decreased hearing ability, tinnitus, and hyperacusis. Second, since the vestibular system is located near the audiological system, it would be a worthy notification if patients complained of vestibular symptoms. Third, clinicians should consider an ENT doctor referral if their patients’ hearing impairment is less likely related to ototoxicity of a specific drug. Finally, although subjectively, clinicians should consider the option of ENT referral if their patients often ignore the clinician’s verbal voice.

### 4.9. To Resolve the Gap Between RA and Audiological Dysfunction

Finally, as addressed before, since there had not been clear evidence linking the physiopathology of audiological dysfunction and RA systematic features, the proposed treatment and examination tools were all based on hypothesis and clinical observation. Therefore, future clinical research directly addressing the physiopathology feature of this disease should be warranted to resolve this gap.

## 5. Conclusions

**Figure 4:**
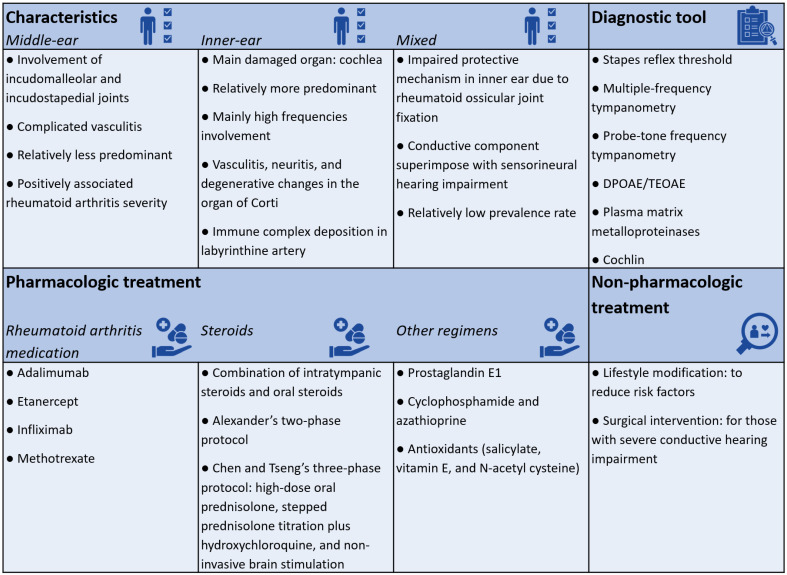
A brief summary of the current systematic review. Figure 4 summarizes the key findings of the current systematic review.

This review article has summarized the current understanding of RA-related hearing impairment (Figure 4). Early detection could significantly improve prognosis. Certain audiometric tests may assist in the early identification of hearing impairment in patients with RA. However, despite statistically significant findings of hearing impairment in both the middle ear and inner ear at specific frequencies among patients with RA, these impairments may not always meet the clinical thresholds for hearing loss [56]. Therefore, despite the lack of awareness of hearing loss, patients with RA should undergo regular hearing assessments to potentially mitigate and prevent future deterioration of their hearing abilities.

## Figures and Tables

**Table 1:** Subtypes of rheumatoid arthritis-related hearing impairment.

Subtype	Characteristics	Physiopathology	Examination
**Middle-ear involvement**	(1) Prevalence rate: 1.9% to 24.3%	(1) Otosclerosis and damage in incudomalleolar and incudostapedial joints (2) Decreased stability of ligamentous anchorage (3) Disk material dissolution with pannus-like tissue formation (4) Rheumatoid nodules formation	(1) Significantly increased ipsilateral stapes reflex threshold value (up to 1.0 kHz) (2) Multiple-frequency tympanometry (from 250 to 2000 Hz) (3) Higher probe-tone frequency tympanometry (4) Plasma matrix metalloproteinases
**Inner-ear involvement**	(1) Prevalence rate: 24–72% (2) More prevalent than middle-ear involvement subtype (3) High percentage of bilateral ear involvement (4) High frequencies predominant (to be specific, 8000 Hz)	(1) Disturbance in cochlear microcirculation via the mechanism of active constriction of the capillaries (2) Direct neuritis or degenerative changes in the organ of the Corti (3) Rheumatoid arthritis-related immune complex deposition in the labyrinthine artery	(1) Distortion-product otoacoustic emission (2) Transient evoked otoacoustic emission (3) Average hearing loss value (4) Rheumatoid arthritis-related data (Ritchie Articular Index, C-reactive protein, erythrocyte sedimentation rate, and platelet counts) (5) Plasma matrix metalloproteinases
**Mixed**	(1) Prevalence rate: 10.4% (2) Happen in a later disease stage	(1) Rheumatoid ossicular joint fixation in the middle ear would result in the impaired protective mechanism in the inner ear (2) Conductive component could superimpose with the sensorineural hearing impairment	(1) Those examinations mentioned in middle-ear and inner-ear subtypes
**Treatment for all three subtypes**			
**All three subtypes**	(1) Rheumatoid arthritis medications (2) Vasodilator (3) Antioxidants (sodium salicylate, vitamin E, and N-acetyl cysteine) (4) Steroid [phase 1: high-dose prednisone (60 mg/day) plus ranitidine for 4 weeks; phase 2: forced downward tapering of oral prednisone over 18 weeks] (5) Lifestyle modification (smoke, alcohol, and noise) (6) Surgical intervention (preserve to severe forms of middle-ear involvement subtype and mixed subtype only)		

## Supplementary Materials

The following supporting information can be downloaded at: https://www.mdpi.com/article/10.3390/ijms252413290/s1. [4,5,6,7,8,9,10,11,12,13,14,15,16,17,18,19,20,21,22,23,24,25,26,27,28,29,31,32,33,36,39,40,41,42,43,44,45,46,47,50,51,52,53,56,58,60,61,62,65,66,67,68,73,74,75,76,77,78,79,80,81,82,83,84,91,92,93,94,95,98,100,103,104,106,108,109,110,111,112,116,117,119,125,129,130,131,132,133,134,135,136,137,138,139,140,141,142,143,144,145,146].
