# Supplementary material for: Audiological Features in Patients with Rheumatoid Arthritis: A Systematic Review
Source: Int J Mol Sci. 2024 Dec 11;25(24):13290. doi: 10.3390/ijms252413290 (PMC11676466; doi:10.3390/ijms252413290)
Supplement: Supplementary file 1 [file ijms-25-13290-s001.zip › ijms-3342542-supplementary.pdf]

Table S1: PRISMA 2020 checklist of current systematic review

Section and Topic	Item #	Checklist item	Page where item is reported
**TITLE**			
Title	1	Identify the report as a systematic review.	1
**ABSTRACT**			
Abstract	2	See the PRISMA 2020 for Abstracts checklist.	3
**INTRODUCTION**			
Rationale	3	Describe the rationale for the review in the context of existing knowledge.	5-6
Objectives	4	Provide an explicit statement of the objective(s) or question(s) the review addresses.	5-6
**METHODS**			
Eligibility criteria	5	Specify the inclusion and exclusion criteria for the review and how studies were grouped for the syntheses.	7-8
Information sources	6	Specify all databases, registers, websites, organisations, reference lists and other sources searched or consulted to identify studies. Specify the date when each source was last searched or consulted.	7-8
Search strategy	7	Present the full search strategies for all databases, registers and websites, including any filters and limits used.	7-8
Selection process	8	Specify the methods used to decide whether a study met the inclusion criteria of the review, including how many reviewers screened each record and each report retrieved, whether they worked independently, and if applicable, details of automation tools used in the process.	7-8
Data collection process	9	Specify the methods used to collect data from reports, including how many reviewers collected data from each report, whether they worked independently, any processes for obtaining or confirming data from study investigators, and if applicable, details of automation tools used in the process.	7-8
Data items	10a	List and define all outcomes for which data were sought. Specify whether all results that were compatible with each outcome domain in each study were sought (e.g. for all measures, time points, analyses), and if not, the methods used to decide which results to collect.	7-8
	10b	List and define all other variables for which data were sought (e.g. participant and intervention characteristics, funding sources). Describe any assumptions made about any missing or unclear information.	7-8
Study risk of bias assessment	11	Specify the methods used to assess risk of bias in the included studies, including details of the tool(s) used, how many reviewers assessed each study and whether they worked independently, and if applicable, details of automation tools used in the process.	7-8
Effect measures	12	Specify for each outcome the effect measure(s) (e.g. risk ratio, mean difference) used in the synthesis or presentation of results.	7-8
Synthesis methods	13a	Describe the processes used to decide which studies were eligible for each synthesis (e.g. tabulating the study intervention characteristics and comparing against the planned groups for each synthesis (item #5)).	Not done
	13b	Describe any methods required to prepare the data for presentation or synthesis, such as handling of missing summary statistics, or data conversions.	Not done

Section and Topic	Item #	Checklist item	Page where item is reported
	13c	Describe any methods used to tabulate or visually display results of individual studies and syntheses.	Not done
	13d	Describe any methods used to synthesize results and provide a rationale for the choice(s). If meta-analysis was performed, describe the model(s), method(s) to identify the presence and extent of statistical heterogeneity, and software package(s) used.	Not done
	13e	Describe any methods used to explore possible causes of heterogeneity among study results (e.g. subgroup analysis, meta-regression).	Not done
	13f	Describe any sensitivity analyses conducted to assess robustness of the synthesized results.	Not done
Reporting bias assessment	14	Describe any methods used to assess risk of bias due to missing results in a synthesis (arising from reporting biases).	7-8
Certainty assessment	15	Describe any methods used to assess certainty (or confidence) in the body of evidence for an outcome.	7-8
**RESULTS**			
Study selection	16a	Describe the results of the search and selection process, from the number of records identified in the search to the number of studies included in the review, ideally using a flow diagram.	9-11
	16b	Cite studies that might appear to meet the inclusion criteria, but which were excluded, and explain why they were excluded.	9-11
Study characteristics	17	Cite each included study and present its characteristics.	9-11
Risk of bias in studies	18	Present assessments of risk of bias for each included study.	9-11
Results of individual studies	19	For all outcomes, present, for each study: (a) summary statistics for each group (where appropriate) and (b) an effect estimate and its precision (e.g. confidence/credible interval), ideally using structured tables or plots.	9-21
Results of syntheses	20a	For each synthesis, briefly summarise the characteristics and risk of bias among contributing studies.	Not done
	20b	Present results of all statistical syntheses conducted. If meta-analysis was done, present for each the summary estimate and its precision (e.g. confidence/credible interval) and measures of statistical heterogeneity. If comparing groups, describe the direction of the effect.	Not done
	20c	Present results of all investigations of possible causes of heterogeneity among study results.	Not done
	20d	Present results of all sensitivity analyses conducted to assess the robustness of the synthesized results.	Not done
Reporting biases	21	Present assessments of risk of bias due to missing results (arising from reporting biases) for each synthesis assessed.	9-18
Certainty of evidence	22	Present assessments of certainty (or confidence) in the body of evidence for each outcome assessed.	9-18
**DISCUSSION**			
Discussion	23a	Provide a general interpretation of the results in the context of other evidence.	9-18
	23b	Discuss any limitations of the evidence included in the review.	9-18
	23c	Discuss any limitations of the review processes used.	9-18

Section and Topic	Item #	Checklist item	Page where item is reported
	23d	Discuss implications of the results for practice, policy, and future research.	19
**OTHER INFORMATION**			
Registration and protocol	24a	Provide registration information for the review, including register name and registration number, or state that the review was not registered.	4
	24b	Indicate where the review protocol can be accessed, or state that a protocol was not prepared.	4
	24c	Describe and explain any amendments to information provided at registration or in the protocol.	4
Support	25	Describe sources of financial or non-financial support for the review, and the role of the funders or sponsors in the review.	20
Competing interests	26	Declare any competing interests of review authors.	20
Availability of data, code and other materials	27	Report which of the following are publicly available and where they can be found: template data collection forms; data extracted from included studies; data used for all analyses; analytic code; any other materials used in the review.	20

The current checklist followed the latest PRISMA 2020 guideline [1].

Table S2: Keyword and search results in each database

Database	Keyword	Filter	Date*	Result
PubMed	(rheumatoid arthritis OR rheumatoid factor) AND (hearing loss OR sensorineural hearing loss OR SNHL OR audiology OR tinnitus OR vertigo OR vestibular OR dizziness)	N/A	2024/6/21	521
Embase	(rheumatoid arthritis OR rheumatoid factor) AND (hearing loss OR sensorineural hearing loss OR SNHL OR audiology OR tinnitus OR vertigo OR vestibular OR dizziness)	N/A	2024/6/21	3826
ClinicalKey	(rheumatoid arthritis OR rheumatoid factor) AND (hearing loss OR sensorineural hearing loss OR SNHL OR audiology OR tinnitus OR vertigo OR vestibular OR dizziness)	N/A	2024/6/21	639
Web of Science	(rheumatoid arthritis OR rheumatoid factor) AND (hearing loss OR sensorineural hearing loss OR SNHL OR audiology OR tinnitus OR vertigo OR vestibular OR dizziness)	N/A	2024/6/21	395
ScienceDirect on-line	(rheumatoid arthritis) AND (hearing loss OR audiology OR vestibular OR vertigo)	N/A	2024/6/21	9022

*: initial search date on 2024/1/19, final update on 2024/6/21

Abbreviation: N/A: not applied

Table S3: Excluded studies and reason

Reason	Numbers	References
Review article	5	[2-6]
Animal study	1	[7]
Meta-analysis	2	[8,9]
Not specific related to rheumatoid arthritis	14	[10-23]
Not related to audiology dysfunction	6	[24-29]

Table S4: Newcastle-Ottawa Scale for Observational trial

Study	Selection				Comparability	Exposure			Total
	Case definition	Representativeness	Control selection	Control definition	Comparability	Ascertainment	Same method	Non-Response rate	Summary
Almasi, S. (2023)[30]	*	*	*	*	*	*			6*
Effat, K.G. (2023)[31]	*	*	*	*	*	*			6*
Torere, B.E. (2023)[32]	*	*							2*
Tsuchida, Y. (2022)[33]	*	*							2*
Li, H. (2021)[34]	*	*	*	*	*	*			6*
Li, H.H. (2021)[35]	*	*	*	*	*	*			6*
Narayan, M. (2021)[36]	*	*							2*
Wang, G. (2021)[37]	*	*	*	*	*	*	*		7*
Xie, S. (2020)[38]	*	*	*	*	*				5*
Jeong, J. (2019)[39]	*	*	*	*	*				5*

Kiakojuri, K. (2019)[40]	*	*	*	*	*	*			6*
Lee, S.Y. (2019)[41]	*	*	*	*	*	*	*		7*
Nasution, M.E.S. (2019)[42]	*	*	*	*	*	*			6*
Tsirves, G.K. (2019)[43]	*	*	*	*	*	*			6*
Galarza-Delgado, D.A. (2018)[44]	*	*	*	*	*	*		*	7*
Huang, C.M. (2018)[45]	*	*	*	*	*	*		*	7*
Ahmadzadeh, A. (2017)[46]	*	*	*	*	*	*			6*
Rahne, T. (2017)[47]	*	*				*			3*
Jeong, H. (2016)[48]	*	*				*			3*
Lasso de la Vega, M. (2016)[49]	*	*	*	*	*	*			6*
Lobo, F.S. (2016)[50]	*	*	*	*	*				5*

Macias-Reyes, H. (2016)[51]	*	*				*	3*
Yildirim, A. (2016)[52]	*	*	*	*	*		5*
Trevino-Gonzalez, J.L. (2015)[53]	*	*				*	3*
Gazeau, P. (2014)[54]	*	*					2*
Milisavljevic, D. (2014)[55]	*	*					2*
Ozkiris, M. (2014)[56]	*	*	*	*	*		5*
Pascual-Ramos, V. (2014)[57]	*	*				*	3*
Toktas, H. (2014)[58]	*	*					2*
Melikoglu, M.A. (2013)[59]	*	*					2*
Ashok Murthy, V. (2012)[60]	*					*	2*
Lee, L.Y. (2012)[61]	*					*	2*

Pascual-Ramos, V. (2012)[62]	*	*				*	3*
Alonso, L. (2011)[63]	*	*			*	*	4*
Gazquez, I. (2011)[64]	*	*				*	3*
Tavernier, L. (2011)[65]	*					*	2*
Baradaranfar, M.H. (2010)[66]	*	*	*	*	*	*	6*
Morovic Vergles, J. (2010)[67]	*					*	2*
Dikici, O. (2009)[68]	*	*	*	*	*	*	6*
Murdin, L. (2008)[69]	*	*				*	3*
Bayazit, Y.A. (2007)[70]	*	*	*	*	*	*	6*
Garcia Callejo, F.J. (2007)[71]	*	*	*	*	*	*	6*
Halligan, C.S. (2006)[72]	*	*	*	*	*	*	6*

Salvinelli, F. (2006)[73]	*	*	*	*	*	*	6*
Takatsu, M. (2005)[74]	*	*	*	*	*	*	6*
Ozturk, A. (2004)[75]	*	*	*	*	*	*	6*
Salvinelli, F. (2004)[76]	*	*				*	3*
Salvinelli, F. (2004)[77]	*	*	*	*	*	*	6*
Ozcan, M. (2002)[78]	*	*	*	*	*	*	6*
Poorey, V.K. (2001)[79]	*	*	*	*	*	*	6*
Raut, V.V. (2001)[80]	*	*	*	*	*	*	6*
Seckin, U. (2000)[81]	*					*	2*
Frade, C. (1998)[82]	*	*				*	3*
Colletti, V. (1997)[83]	*	*	*	*		*	5*

Kastanioudakis, I. (1995)[84]	*	*			*	3*
Etherington, J. (1994)[85]	*				*	2*
Mukerji, B. (1994)[86]	*				*	2*
Hamidou, M. (1990)[87]	*				*	2*
Kakani, R.S. (1990)[88]	*	*	*	*	*	5*
Magaro, M. (1990)[89]	*	*			*	3*
Hamidou, M. (1989)[90]	*				*	2*
Nores, J.M. (1989)[91]	*				*	2*
Ferrara, P. (1988)[92]	*	*			*	3*
Nores, J.M. (1988)[93]	*				*	2*
Elwany, S. (1986)[94]	*	*			*	3*

Reiter, D. (1980)[95]	*	*	*	*	*	5*
Walek, H. (1980)[96]	*	*			*	3*
Rosenberg, J.N. (1978)[97]	*	*	*	*	*	5*
Kremer, D. (1975)[98]	*				*	2*
Goodwill, C.J. (1972)[99]	*	*			*	3*
Heyworth, T. (1972)[100]	*	*			*	3*
Goodwill, C.J. (1971)	*	*			*	3*
Capeman, W.S. (1963)[101]	*				*	2*

* indicated this study have a good performance in this item

Reference list of supplement table:

1. Page, M.J.; McKenzie, J.E.; Bossuyt, P.M.; Boutron, I.; Hoffmann, T.C.; Mulrow, C.D.; Shamseer, L.; Tetzlaff, J.M.; Akl, E.A.; Brennan, S.E.; et al. The PRISMA 2020 statement: an updated guideline for reporting systematic reviews. *Bmj* **2021**, *372*, n71, doi:10.1136/bmj.n71.
2. Khoza-Shangase, K.; Riva, R. Hearing Function in Adults with Rheumatoid Arthritis: A Scoping Review for Preventive Audiology Planning. *Indian J Otolaryngol Head Neck Surg* **2022**, *74*, 3965-3976, doi:10.1007/s12070-021-02747-x.
3. Emamifar, A.; Hansen, I.M.J. An update on hearing impairment in patients with rheumatoid arthritis. *J Otol* **2018**, *13*, 1-4, doi:10.1016/j.joto.2017.10.002.
4. Caulley, L.; Quimby, A.; Karsh, J.; Ahrari, A.; Tse, D.; Kontorinis, G. Autoimmune arthritis in Meniere's disease: A systematic review of the literature. *Semin Arthritis Rheum* **2018**, *48*, 141-147, doi:10.1016/j.semarthrit.2017.11.008.
5. Emamifar, A.; Bjoerndal, K.; Hansen, I.M. Is Hearing Impairment Associated with Rheumatoid Arthritis? A Review. *Open Rheumatol J* **2016**, *10*, 26-32, doi:10.2174/1874312901610010026.
6. Barna, B.P.; Hughes, G.B. Autoimmunity and otologic disease: clinical and experimental aspects. *Clin Lab Med* **1988**, *8*, 385-398.
7. Chen, R.; Schwander, M.; Barbe, M.F.; Chan, M.M. Ossicular Bone Damage and Hearing Loss in Rheumatoid Arthritis: A Correlated Functional and High Resolution Morphometric Study in Collagen-Induced Arthritic Mice. *PloS one* **2016**, *11*, e0164078, doi:10.1371/journal.pone.0164078.
8. Li, X.; Cao, Z.; Chen, F.; Yang, D.; Zhao, F. Sensorineural Hearing Loss in Autoimmune Diseases: A Systematic Review and Meta-analysis. *J Int Adv Otol* **2023**, *19*, 277-282, doi:10.5152/iao.2023.22991.
9. Chaitidis, N.; Theocharis, P.; Festas, C.; Aritzi, I. Association of rheumatoid arthritis with hearing loss: a systematic review and meta-analysis. *Rheumatol Int* **2020**, *40*, 1771-1779, doi:10.1007/s00296-020-04609-1.
10. Baradaranfar, M.; Dadgarnia, M.; Zand, V.; Vaziribozorg, S.; Mirzade, F.S.; Mirzade, M. The Role of Immunological Factors on Sudden Sensoryneural Hearing Loss. *Iran J Otorhinolaryngol* **2018**, *30*, 219-223.

11. Rajati, M.; Saghafi, M.; Rafatpanah, H.; Rasoulian, B.; Irani, S.; Soltankhah, M. Immunology-Rheumatology Approach to Sudden Sensorineural Hearing Loss. *Curr Rheumatol Rev* **2018**, *14*, 70-73, doi:10.2174/1573397112666161029221905.
12. Cho, C.H.; Jung, B.S.; Jung, J.H.; Lee, J.H.; Lee, J.H. Expression of autoantibodies in patients with sudden sensorineural hearing loss. *Ann Otol Rhinol Laryngol* **2013**, *122*, 131-134, doi:10.1177/000348941312200209.
13. Conway, R.; Khan, S.; Foley-Nolan, D. Use of adalimumab in treatment of autoimmune sensorineural hearing loss: a word of caution. *J Rheumatol* **2011**, *38*, 176; author reply 176, doi:10.3899/jrheum.100593.
14. Hervier, B.; Bordure, P.; Audrain, M.; Calais, C.; Masseau, A.; Hamidou, M. Systematic screening for nonspecific autoantibodies in idiopathic sensorineural hearing loss: no association with steroid response. *Otol Neurotol* **2010**, *31*, 687-690, doi:10.1097/MAO.0b013e3181dd13cc.
15. Dayal, V.S.; Ellman, M.; Sweiss, N. Autoimmune inner ear disease: clinical and laboratory findings and treatment outcome. *J Otolaryngol Head Neck Surg* **2008**, *37*, 591-596.
16. Toubi, E.; Ben-David, J.; Kessel, A.; Halas, K.; Sabo, E.; Luntz, M. Immune-mediated disorders associated with idiopathic sudden sensorineural hearing loss. *Ann Otol Rhinol Laryngol* **2004**, *113*, 445-449, doi:10.1177/000348940411300605.
17. Ruckenstein, M.J.; Prasthoffer, A.; Bigelow, D.C.; Von Feldt, J.M.; Kolasinski, S.L. Immunologic and serologic testing in patients with Meniere's disease. *Otol Neurotol* **2002**, *23*, 517-520; discussion 520-511, doi:10.1097/00129492-200207000-00021.
18. Rahman, M.U.; Poe, D.S.; Choi, H.K. Etanercept therapy for immune-mediated cochleovestibular disorders: preliminary results in a pilot study. *Otol Neurotol* **2001**, *22*, 619-624, doi:10.1097/00129492-200109000-00010.
19. Takahashi, M.; Sakata, A.; Unno, T.; Hokunan, K.; Shigyo, H. [Immunological abnormalities in patients with etiology unknown sensorineural hearing loss]. *Nihon Jibiinkoka Gakkai kaiho* **1998**, *101*, 1260-1265, doi:10.3950/jibiinkoka.101.10_1260.
20. Yoshida, Y.; Yamauchi, S.; Shinkawa, A.; Horiuchi, M.; Sakai, M. Immunological and virological study of sudden deafness. *Auris Nasus Larynx* **1996**, *23*, 63-68, doi:10.1016/s0385-8146(96)80010-1.
21. Tumiati, B.; Casoli, P. Sudden sensorineural hearing loss and anticardiolipin antibody. *Am J Otolaryngol* **1995**, *16*, 220, doi:10.1016/0196-0709(95)90109-4.

22. Hughes, G.B.; Barna, B.P.; Kinney, S.E.; Calabrese, L.H.; Nalepa, N.J. Clinical diagnosis of immune inner-ear disease. *Laryngoscope* **1988**, *98*, 251-253, doi:10.1288/00005537-198803000-00001.
23. Gussen, R. Atypical ossicle joint lesions in rheumatoid arthritis with sicca syndrome (Sjogren syndrome). *Arch Otolaryngol* **1977**, *103*, 284-286, doi:10.1001/archotol.1977.00780220078009.
24. Kent, A.E.; Gurses, E.; Karabekiroglu, F.; Genc, A. Balance Impairment in Patients with Rheumatoid Arthritis and Ankylosing Spondylitis. *Curr Rheumatol Rev* **2023**, doi:10.2174/1573397119666230828162611.
25. Zonzini Gaino, J.; Barros Bertolo, M.; Silva Nunes, C.; de Morais Barbosa, C.; Sachetto, Z.; Davitt, M.; de Paiva Magalhaes, E. Disease-related outcomes influence prevalence of falls in people with rheumatoid arthritis. *Ann Phys Rehabil Med* **2019**, *62*, 84-91, doi:10.1016/j.rehab.2018.09.003.
26. Roberts, R.A. Management of Recurrent Vestibular Neuritis in a Patient Treated for Rheumatoid Arthritis. *Am J Audiol* **2018**, *27*, 19-24, doi:10.1044/2017_AJA-17-0090.
27. Heydari, N.; Hajiabolhassani, F.; Fatahi, J.; Movaseghi, S.; Jalaie, S. Vestibular evoked myogenic potentials in patients with rheumatoid arthritis. *Med J Islam Repub Iran* **2015**, *29*, 216.
28. Yilmaz, S.; Erbek, S.; Erbek, S.S.; Ozgirgin, N.; Yucel, E. Abnormal electronystagmography in rheumatoid arthritis. *Auris Nasus Larynx* **2007**, *34*, 307-311, doi:10.1016/j.anl.2006.11.003.
29. King, J.; Young, C.; Highton, J.; Smith, P.F.; Darlington, C.L. Vestibulo-ocular, optokinetic and postural function in humans with rheumatoid arthritis. *Neurosci Lett* **2002**, *328*, 77-80, doi:10.1016/s0304-3940(02)00219-7.
30. Almasi, S.; Mehrabian, F.; Rahbar, N.; Delarestaghi, M.M. Prevalence Rate of Hearing Loss in Patients with Rheumatoid Arthritis. *Advanced biomedical research* **2023**, *12*, 80, doi:10.4103/abr.abr_118_21.
31. Effat, K.G.; Berty, A. Otological symptoms in patients with rheumatoid arthritis of the temporomandibular joint. *Cranio* **2023**, 1-8, doi:10.1080/08869634.2023.2260281.
32. Torere, B.E.; Chittipolu, S.; Alugba, G.; Aiwuyo, H.O.; Kennard, J.L. Sudden-Onset Sensorineural Hearing Loss and Tinnitus in a Patient With Rheumatoid Arthritis: A Case Report and Literature Review. *Cureus* **2023**, *15*, e38739, doi:10.7759/cureus.38739.

33. Tsuchida, Y.; Nagafuchi, Y.; Uehara, T.; Suzuki, H.; Yamada, M.; Kono, M.; Hatano, H.; Shoda, H.; Fujio, K.; Kosaki, K. Rheumatoid arthritis in a patient with compound heterozygous variants in the COL11A2 gene and progressive hearing loss: A case report. *Medicine* **2022**, *101*, e28828, doi:10.1097/MD.0000000000028828.
34. Li, H.; Zhang, M.; Wang, M.; Zhang, S.; Ma, S.; Wang, X. Clinical Feature and Prognosis of Sudden Sensorineural Hearing Loss With Rheumatoid Arthritis. *Otol Neurotol* **2021**, *42*, e267-e271, doi:10.1097/MAO.0000000000002962.
35. Li, H.H.; Livneh, H.; Chen, W.J.; Fan, W.L.; Lu, M.C.; Guo, H.R.; Tsai, T.Y. Effect of Chinese Herbal Medicines on Hearing Loss Risk in Rheumatoid Arthritis Patients: Retrospective Claims Analysis. *Front Med (Lausanne)* **2021**, *8*, 683211, doi:10.3389/fmed.2021.683211.
36. Narayan, M.; Sreedharan, S.; Pai, R.; Shenoy, S. Sudden Hearing Loss and Multiple Cranial Nerve Palsies in Autoimmune disease: A Case Report. *Iran J Otorhinolaryngol* **2021**, *33*, 45-48, doi:10.22038/ijorl.2020.47096.2608.
37. Wang, G.; Deng, G.; Liang, J.; Zhao, Z. [Clinical characteristics and prognosis of sudden sensorineural hearing loss with rheumatoid arthritis]. *Lin Chuang Er Bi Yan Hou Tou Jing Wai Ke Za Zhi* **2021**, *35*, 9-13, doi:10.13201/j.issn.2096-7993.2021.01.002.
38. Xie, S.; Ning, H.; She, Y.; Jing, Q.; Jiang, Q.; Zhang, Y.; Mei, L.; Feng, Y.; Wu, X. Effect of systemic lupus erythematosus and rheumatoid arthritis on sudden sensorineural hearing loss. *Laryngoscope* **2020**, *130*, 2475-2480, doi:10.1002/lary.28455.
39. Jeong, J.; Lim, H.; Lee, K.; Hong, C.E.; Choi, H.S. High Risk of Sudden Sensorineural Hearing Loss in Several Autoimmune Diseases according to a Population-Based National Sample Cohort Study. *Audiol Neurootol* **2019**, *24*, 224-230, doi:10.1159/000502677.
40. Kiakojuri, K.; Yousef Ghahari, B.; Soltanparast, S.; Monadi, M. Hearing status in patients with rheumatoid arthritis. *Caspian J Intern Med* **2019**, *10*, 447-451, doi:10.22088/cjim.10.4.447.
41. Lee, S.Y.; Kong, I.G.; Oh, D.J.; Choi, H.G. Increased risk of sudden sensory neural hearing loss in patients with rheumatoid arthritis: a longitudinal follow-up study using a national sample cohort. *Clin Rheumatol* **2019**, *38*, 683-689, doi:10.1007/s10067-018-4333-6.
42. Nasution, M.E.S.; Haryuna, T.S.H. Elevated matrix metalloproteinase-3 level may affect hearing function in patients with rheumatoid arthritis. *J Chin Med Assoc* **2019**, *82*, 272-276, doi:10.1097/JCMA.0000000000000036.
43. Tsirves, G.K.; Voulgari, P.V.; Pelechas, E.; Asimakopoulos, A.D.; Drosos, A.A. Cochlear involvement in patients with systemic autoimmune rheumatic diseases: a clinical and laboratory comparative study. *Eur Arch Otorhinolaryngol* **2019**, *276*, 2419-2426, doi:10.1007/s00405-

019-05487-5.
44. Galarza-Delgado, D.A.; Villegas Gonzalez, M.J.; Riega Torres, J.; Soto-Galindo, G.A.; Mendoza Flores, L.; Trevino Gonzalez, J.L. Early hearing loss detection in rheumatoid arthritis and primary Sjogren syndrome using extended high frequency audiometry. *Clin Rheumatol* **2018**, *37*, 367-373, doi:10.1007/s10067-017-3959-0.
45. Huang, C.M.; Chen, H.J.; Huang, P.H.; Tsay, G.J.; Lan, J.L.; Sung, F.C. Retrospective cohort study on risk of hearing loss in patients with rheumatoid arthritis using claims data. *BMJ Open* **2018**, *8*, e018134, doi:10.1136/bmjopen-2017-018134.
46. Ahmadzadeh, A.; Daraei, M.; Jalessi, M.; Peyvandi, A.A.; Amini, E.; Ranjbar, L.A.; Daneshi, A. Hearing status in patients with rheumatoid arthritis. *J Laryngol Otol* **2017**, *131*, 895-899, doi:10.1017/S0022215117001670.
47. Rahne, T.; Clauss, F.; Plontke, S.K.; Keysser, G. Prevalence of hearing impairment in patients with rheumatoid arthritis, granulomatosis with polyangiitis (GPA, Wegener's granulomatosis), or systemic lupus erythematosus. *Clin Rheumatol* **2017**, *36*, 1501-1510, doi:10.1007/s10067-017-3651-4.
48. Jeong, H.; Chang, Y.S.; Baek, S.Y.; Kim, S.W.; Eun, Y.H.; Kim, I.Y.; Lee, J.; Koh, E.M.; Cha, H.S. Evaluation of Audiometric Test Results to Determine Hearing Impairment in Patients with Rheumatoid Arthritis: Analysis of Data from the Korean National Health and Nutrition Examination Survey. *PloS one* **2016**, *11*, e0164591, doi:10.1371/journal.pone.0164591.
49. Lasso de la Vega, M.; Villarreal, I.M.; Lopez-Moya, J.; Garcia-Berrocal, J.R. Examination of Hearing in a Rheumatoid Arthritis Population: Role of Extended-High-Frequency Audiometry in the Diagnosis of Subclinical Involvement. *Scientifica (Cairo)* **2016**, *2016*, 5713283, doi:10.1155/2016/5713283.
50. Lobo, F.S.; Dossi, M.O.; Batista, L.; Shinzato, M.M. Hearing impairment in patients with rheumatoid arthritis: association with anti-citrullinated protein antibodies. *Clin Rheumatol* **2016**, *35*, 2327-2332, doi:10.1007/s10067-016-3278-x.
51. Macias-Reyes, H.; Duran-Barragan, S.; Cardenas-Contreras, C.R.; Chavez-Martin, C.G.; Gomez-Banuelos, E.; Navarro-Hernandez, R.E.; Yanowsky-Gonzalez, C.O.; Gonzalez-Lopez, L.; Gamez-Nava, J.I.; Vazquez-Del Mercado, M. Sensorineural Hearing Impairment and Subclinical Atherosclerosis in Rheumatoid Arthritis Patients Without Traditional Cardiovascular Risk Factors. *Arch Rheumatol* **2016**, *31*, 208-214, doi:10.5606/ArchRheumatol.2016.5739.

52. Yildirim, A.; Surucu, G.; Dogan, S.; Karabiber, M. Relationship between disease activity and hearing impairment in patients with rheumatoid arthritis compared with controls. *Clin Rheumatol* **2016**, *35*, 309-314, doi:10.1007/s10067-015-3129-1.
53. Trevino-Gonzalez, J.L.; Villegas-Gonzalez, M.J.; Munoz-Maldonado, G.E.; Montero-Cantu, C.A.; Nava-Zavala, A.H.; Garza-Elizondo, M.A. [Subclinical sensorineural hearing loss in female patients with rheumatoid arthritis]. *Cir Cir* **2015**, *83*, 364-370, doi:10.1016/j.circir.2015.05.026.
54. Gazeau, P.; Saraux, A.; Devauchelle-Pensec, V.; Cornec, D. Long-term efficacy of infliximab in autoimmune sensorineural hearing loss associated with rheumatoid arthritis. *Rheumatology (Oxford)* **2014**, *53*, 1715-1716, doi:10.1093/rheumatology/keu025.
55. Milisavljevic, D.; Stankovic, I.; Stankovic, M. Is the treatment of hearing loss in rheumatoid arthritis effective? *Hippokratia* **2014**, *18*, 288.
56. Ozkiris, M.; Kapusuz, Z.; Gunaydin, I.; Kubilay, U.; Pirti, I.; Saydam, L. Does rheumatoid arthritis have an effect on audiovestibular tests? *Eur Arch Otorhinolaryngol* **2014**, *271*, 1383-1387, doi:10.1007/s00405-013-2551-8.
57. Pascual-Ramos, V.; Contreras-Yanez, I.; Rivera-Hoyos, P.; Enriquez, L.; Ramirez-Anguiano, J. Cumulative disease activity predicts incidental hearing impairment in patients with rheumatoid arthritis (RA). *Clin Rheumatol* **2014**, *33*, 315-321, doi:10.1007/s10067-014-2485-6.
58. Toktas, H.; Okur, E.; Dundar, U.; Dikici, A.; Kahveci, O.K. Infliximab has no apparent effect in the inner ear hearing function of patients with rheumatoid arthritis and ankylosing spondylitis. *Clin Rheumatol* **2014**, *33*, 1481-1487, doi:10.1007/s10067-014-2625-z.
59. Melikoglu, M.A.; Senel, K. Sudden hearing loss in a patient with rheumatoid arthritis; a case report and review of the literature. *Acta reumatologica portuguesa* **2013**, *38*, 138-139.
60. Ashok Murthy, V.; Mohan Kumar, J. Rheumatoid factor and hearing loss. *Indian J Otolaryngol Head Neck Surg* **2012**, *64*, 364-365, doi:10.1007/s12070-011-0401-9.
61. Lee, L.Y.; Akhtar, M.M.; Kirresh, O.; Gibson, T. Interstitial keratitis and sensorineural hearing loss as a manifestation of rheumatoid arthritis: clinical lessons from a rare complication. *BMJ case reports* **2012**, *2012*, doi:10.1136/bcr-2012-007324.
62. Pascual-Ramos, V.; Contreras-Yanez, I.; Enriquez, L.; Valdes, S.; Ramirez-Anguiano, J. Hearing impairment in a tertiary-care-level population of Mexican rheumatoid arthritis patients. *J Clin Rheumatol* **2012**, *18*, 393-398, doi:10.1097/RHU.0b013e31827732d3.

63. Alonso, L.; Gutierrez-Farfan, I.; Pena-Ayala, A.; Perez-Bastidas, M.E.; Espinosa, R. Clinical significance of auditive involvement in rheumatoid arthritis: a case-control study. *ISRN Rheumatol* **2011**, *2011*, 208627, doi:10.5402/2011/208627.
64. Gazquez, I.; Soto-Varela, A.; Aran, I.; Santos, S.; Batuecas, A.; Trinidad, G.; Perez-Garrigues, H.; Gonzalez-Oller, C.; Acosta, L.; Lopez-Escamez, J.A. High prevalence of systemic autoimmune diseases in patients with Meniere's disease. *PloS one* **2011**, *6*, e26759, doi:10.1371/journal.pone.0026759.
65. Tavernier, L.; Ranfaing, E. Rheumatoid arthritis involved in the ear drum. *Otol Neurotol* **2011**, *32*, e5-6, doi:10.1097/MAO.0b013e3181d2f039.
66. Baradaranfar, M.H.; Doosti, A. A survey of relationship between rheumatoid arthritis and hearing disorders. *Acta Med Iran* **2010**, *48*, 371-373.
67. Morovic Vergles, J.; Radic, M.; Kovacic, J.; Salamon, L. Successful use of adalimumab for treating rheumatoid arthritis with autoimmune sensorineural hearing loss: two birds with one stone. *J Rheumatol* **2010**, *37*, 1080-1081, doi:10.3899/jrheum.091342.
68. Dikici, O.; Muluk, N.B.; Tosun, A.K.; Unlusoy, I. Subjective audiological tests and transient evoked otoacoustic emissions in patients with rheumatoid arthritis: analysis of the factors affecting hearing levels. *Eur Arch Otorhinolaryngol* **2009**, *266*, 1719-1726, doi:10.1007/s00405-009-0975-y.
69. Murdin, L.; Patel, S.; Walmsley, J.; Yeoh, L.H. Hearing difficulties are common in patients with rheumatoid arthritis. *Clin Rheumatol* **2008**, *27*, 637-640, doi:10.1007/s10067-007-0802-z.
70. Bayazit, Y.A.; Yilmaz, M.; Gunduz, B.; Altinyay, S.; Kemaloglu, Y.K.; Onder, M.; Gurer, M.A. Distortion product otoacoustic emission findings in Behcet's disease and rheumatoid arthritis. *ORL J Otorhinolaryngol Relat Spec* **2007**, *69*, 233-238, doi:10.1159/000101544.
71. Garcia Callejo, F.J.; Conill Tobias, N.; Munoz Fernandez, N.; de Paula Vernetta, C.; Alonso Castaneira, I.; Marco Algarra, J. [Hearing impairment in patients with rheumatoid arthritis]. *Acta Otorrinolaringol Esp* **2007**, *58*, 232-238.
72. Halligan, C.S.; Bauch, C.D.; Brey, R.H.; Achenbach, S.J.; Bamlet, W.R.; McDonald, T.J.; Matteson, E.L. Hearing loss in rheumatoid arthritis. *Laryngoscope* **2006**, *116*, 2044-2049, doi:10.1097/01.mlg.0000241365.54017.32.
73. Salvinelli, F.; D'Ascanio, L.; Casale, M.; Vadacca, M.; Rigon, A.; Afeltra, A. Auditory pathway in rheumatoid arthritis. A comparative study

and surgical perspectives. *Acta Otolaryngol* **2006**, *126*, 32-36, doi:10.1080/00016480510012264.
74. Takatsu, M.; Higaki, M.; Kinoshita, H.; Mizushima, Y.; Koizuka, I. Ear involvement in patients with rheumatoid arthritis. *Otol Neurotol* **2005**, *26*, 755-761, doi:10.1097/01.mao.0000178138.19848.bd.
75. Ozturk, A.; Yalcin, S.; Kaygusuz, I.; Sahin, S.; Gok, U.; Karlidag, T.; Ardicoglu, O. High-frequency hearing loss and middle ear involvement in rheumatoid arthritis. *Am J Otolaryngol* **2004**, *25*, 411-417, doi:10.1016/j.amjoto.2004.06.001.
76. Salvinelli, F.; Cancilleri, F.; Casale, M.; Luccarelli, V.; Di Peco, V.; D'Ascanio, L.; De Martino, A.; Denaro, V. Hearing thresholds in patients affected by rheumatoid arthritis. *Clin Otolaryngol Allied Sci* **2004**, *29*, 75-79, doi:10.1111/j.1365-2273.2004.00783.x.
77. Salvinelli, F.; D'Ascanio, L.; Casale, M. Staging rheumatoid arthritis: what about otoacoustic emissions? *Acta Otolaryngol* **2004**, *124*, 874-875, doi:10.1080/00016480410018205.
78. Ozcan, M.; Karakus, M.F.; Gunduz, O.H.; Tuncel, U.; Sahin, H. Hearing loss and middle ear involvement in rheumatoid arthritis. *Rheumatol Int* **2002**, *22*, 16-19, doi:10.1007/s00296-002-0185-z.
79. Poorey, V.K.; Khatri, R. Study of Auditory function in Rheumatoid Arthritis. *Indian J Otolaryngol Head Neck Surg* **2001**, *53*, 261-263, doi:10.1007/BF02991542.
80. Raut, V.V.; Cullen, J.; Cathers, G. Hearing loss in rheumatoid arthritis. *J Otolaryngol* **2001**, *30*, 289-294, doi:10.2310/7070.2001.19580.
81. Seckin, U.; Ozoran, K.; Ikinciogullari, A.; Borman, P.; Bostan, E.E. Hydroxychloroquine ototoxicity in a patient with rheumatoid arthritis. *Rheumatol Int* **2000**, *19*, 203-204, doi:10.1007/s002960000054.
82. Frade, C.; Martin, C. Diagnostic value of the multifrequency tympanometry in active rheumatoid arthritis. *Auris Nasus Larynx* **1998**, *25*, 131-136, doi:10.1016/s0385-8146(98)00020-0.
83. Colletti, V.; Fiorino, F.G.; Bruni, L.; Biasi, D. Middle ear mechanics in subjects with rheumatoid arthritis. *Audiology* **1997**, *36*, 136-146, doi:10.3109/00206099709071967.
84. Kastanioudakis, I.; Skevas, A.; Danielidis, V.; Tsiakou, E.; Drosos, A.A.; Moustopoulos, M.H. Inner ear involvement in rheumatoid arthritis: a prospective clinical study. *J Laryngol Otol* **1995**, *109*, 713-718, doi:10.1017/s0022215100131135.
85. Etherington, J.; Cooper, A.; Buchanan, N. Antiphospholipid syndrome in rheumatoid arthritis presenting with sensorineural deafness.

Lupus **1994**, *3*, 63-64, doi:10.1177/096120339400300113.
86. Mukerji, B.; Estrem, S.A.; O'Sullivan, F.X. The challenge of sensorineural hearing loss in rheumatoid arthritis. *J Rheumatol* **1994**, *21*, 1753-1757.
87. Hamidou, M.; Morlock, G.; Chalard, R.G.; Cadene, C. [Rheumatoid polyarthritis and deafness. Apropos of a case of oto-arthritis]. *Rev Rhum Mal Osteoartic* **1990**, *57*, 223-224.
88. Kakani, R.S.; Mehra, Y.N.; Deodhar, S.D.; Mann, S.B.; Mehta, S. Audiovestibular functions in rheumatoid arthritis. *J Otolaryngol* **1990**, *19*, 100-102.
89. Magaro, M.; Zoli, A.; Altomonte, L.; Mirone, L.; Corvino, G.; Di Girolamo, S.; Giacomini, P.; Alessandrini, M. Sensorineural hearing loss in rheumatoid arthritis. *Clinical and experimental rheumatology* **1990**, *8*, 487-490.
90. Hamidou, M.; Morlock, G.; Cadene, C.; Chalard, R.G. [Rheumatoid oto-arthritis: a rare cause of deafness]. *Presse Med* **1989**, *18*, 841.
91. Nores, J.M.; Bonfils, P. [Rheumatoid polyarthritis and autoimmune deafness. Apropos of a case]. *Ann Med Interne (Paris)* **1989**, *140*, 239-240.
92. Ferrara, P.; Modica, A.; Adelfio, M.; Salli, L.; Pappalardo, A. [Audio-vestibular changes in patients with rheumatoid arthritis]. *Minerva Med* **1988**, *79*, 1043-1047.
93. Nores, J.M.; Bonfils, P. Rheumatoid arthritis and auto-immune hearing loss. A case study. *Clin Rheumatol* **1988**, *7*, 520-521.
94. Elwany, S.; el Garf, A.; Kamel, T. Hearing and middle ear function in rheumatoid arthritis. *J Rheumatol* **1986**, *13*, 878-881.
95. Reiter, D.; Konkle, D.F.; Myers, A.R.; Schimmer, B.; Sugar, J.O. Middle ear immittance in rheumatoid arthritis. *Arch Otolaryngol* **1980**, *106*, 114-117, doi:10.1001/archotol.1980.00790260046013.
96. Walek, H.; Fritze, W.; Kolarz, G. [Possible involvement of the auditory system in rheumatoid arthritis]. *Zeitschrift fur Rheumatologie* **1980**, *39*, 91-94.
97. Rosenberg, J.N.; Moffat, D.A.; Ramsden, R.T.; Gibson, W.P.; Booth, J.B. Middle ear function in rheumatoid arthritis. *Ann Rheum Dis* **1978**, *37*, 522-524, doi:10.1136/ard.37.6.522.
98. Kremer, D.; Hood, W.G.; Nuki, G. Sudden deafness with serosanguinous middle ear effusions in a patient with rheumatoid arthritis. *Br J*

Clin Pract **1975**, *29*, 347-348.
99. Goodwill, C.J.; Lord, I.J.; Jones, R.P. Hearing in rheumatoid arthritis. A clinical and audiometric survey. *Ann Rheum Dis* **1972**, *31*, 170-173, doi:10.1136/ard.31.3.170.
100. Heyworth, T.; Liyanage, S.P. A pilot survey of hearing loss in patients with rheumatoid arthritis. *Scand J Rheumatol* **1972**, *1*, 81-83, doi:10.3109/03009747209103000.
101. Capeman, W.S. Rheumatoid Oto-Arthritis. *British medical journal* **1963**, *2*, 1526-1527, doi:10.1136/bmj.2.5371.1526-b.
